# Mediating effect of anxiety and depression on the relationship between Attention-deficit/hyperactivity disorder symptoms and smoking/drinking

**DOI:** 10.1038/srep21609

**Published:** 2016-02-29

**Authors:** Lian Tong, Hui-Jing Shi, Zhe Zhang, Yuan Yuan, Zhi-Juan Xia, Xiao-Xiao Jiang, Xu Xiong

**Affiliations:** 1 School of Public Health, Fudan University/Key Laboratory of Public Health Safety, Chinese Ministry of Education, Shanghai, China; 2 Shanghai Public Health School, Shanghai, China; 3 Shanghai Jiao Tong University, Shanghai, China; 4 School of Public Health and Tropical Medicine, Tulane University, New Orleans, USA

## Abstract

Attention-deficit/hyperactivity disorder (ADHD) has been often found to be comorbid with other disorders, including anxiety, depression, and unhealthy behaviors such as drinking alcohol and smoking. These factors were often discussed separately, and the mediating effects of mental health on substance use are unknown. To study the mediating effects of anxiety and depression on the relationship between ADHD and drinking/smoking behaviors, we conducted a cross-sectional study of 1870 college students from Shanghai, China. The Adult ADHD Self-Report Scale (ASRS-v1.1) and Wender Utah Rating Scale (WURS) were used to identify the current and past ADHD. Structural Equation Modeling was carried out to clarify the mediating effect of anxiety and depression on the relationship between core ADHD symptoms and smoking/drinking behaviors. We found that inattention as one of the core symptoms of ADHD was associated with an increased risk of depression as a direct effect, as well as slightly increased risk of smoking/drinking behaviors by an indirect effect of depression. Hyperactivity-impulsivity, as another core symptom of ADHD had a robust impact on smoking and drinking behaviors, while being mediated by anxiety and depression. In conclusion, anxiety and depression was associated with further increased risk behaviors of smoking/drinking alcohol among those students with ADHD.

Attention-deficit/hyperactivity disorder (ADHD) is a common neuropsychiatric disorder among children and adults, worldwide^1^. A large number of studies exists detailing ADHD in early adolescence, but information concerning ADHD in late adolescence or young adulthood is relatively scant. Several studies have attempted to identify the percentage of college students who report clinically significant levels of ADHD symptoms. Collectively, these studies suggest that there are increasing numbers of college students with ADHD, with 2-8% rate of ADHD symptoms^2^,^3^. A cross-culture study of ADHD symptoms among college students in China and the United States revealed that 4.4% of American students and 7.8% of Chinese students had significant ADHD symptoms in their current functioning^4^. ADHD has often been found to be comorbid with other disorders, including both internalizing (13% to 51%) and externalizing disorders (43% to 93%)^5^. Young persons with ADHD are more likely to smoke cigarettes, consume alcohol and take illicit drugs^6^,^7^. The prevalence rate for comorbid substance abuse disorder in adult ADHD patients is as high as 58%^8^. A cross-sectional study of comorbidities found 33% of alcohol abuse and 37% of depression among clinically referred adults with ADHD^9^. College students with ADHD were 2.5-3.5 times more likely to smoke cigarettes than students without ADHD^10^.

ADHD also has a close relationship with mental health. For instance, in an ADHD treatment-seeking group, 18% had major depressive disorder and 32% had general anxiety disorder^11^. Relatively few studies have investigated the psychological functioning of college students with ADHD. A limited number studies suggest that college students with ADHD had more symptoms of depression than those without the disorder^12^,^13^. For example, Richards *et al.* (1999) compared college students with and without ADHD on the self-report symptom inventory (Symptom checklist 90, SCL-90-R) and found the overall score was significantly higher for students with ADHD than for non-ADHD students^14^. The ADHD group reported significantly higher ratings on several subscales including anxiety, and depression. A study carried out in Taiwan had similar findings, indicating that ADHD in young men is associated with more depressive symptoms and anxious symptoms^15^.

As mentioned above, ADHD comorbid with substance use and anxiety and depression were broadly discussed. Among these, anxiety and depression were closely related with substance use, but the results were mixed. Whether substance use is causally associated with depression and anxiety, or opposite causality, consistent and robust genetic polymorphisms had been found by an approach of Mendelian randomization. It suggested that in fact this relationship is more consistent with a self-medication hypothesis than with tobacco use being a risk factor for depression and anxiety^16^. A self-medication hypothesis proposed that anxiety and depression onset before smoking. Because nicotine has been shown to improve performance and decrease distractibility^17^,^18^, the idea that smoking represents a form of self-medication of ADHD symptomatology^19^. Another study by Mendelian randomization suggested that smoking is not a cause of anxiety and depression^20^. A 10-year follow-up study also found that there is a significant prospective risk posed by baseline mental health disorder, like certain mood and anxiety disorder, for the onset of nicotine, alcohol dependence over the follow-up period^21^. Therefore, in the present study, anxiety and depression was supposed to be causally related with smoking and drinking.

One study suggested that anxiety and depression is closely related with substance use independent of ADHD^22^, but the role of anxiety and depression on the relationship with ADHD and substance use is unknown. Among the limited studies that explored the relationship among ADHD, substance use and anxiety and depression, the effect of anxiety and depression was often excluded from the analysis models. For instance, a study based on adolescents indicated that ADHD symptoms may predict substance use independent of other psychiatric and behavioral problems^7^. A recent study suggested that ADHD symptoms predicted smoking and drinking alcohol when controlling for anxiety and depression in adolescents^23^. However, the mediating effect is unclear of mental health such as anxiety and depression on the relationship between ADHD and smoking/drinking alcohol is unclear. Therefore, our first hypothesis is that ADHD symptoms increase the risk of smoking/drinking alcohol, and the relationship may be intensified by anxiety and depression in college students.

ADHD symptoms may change over the age span. Hyperactive motor behavior typically observed in childhood diminishes among adolescents and young adults, and is replaced by a sense of mental or internal restlessness, according to another study^24^. Among college students with ADHD, inattentive symptoms tend to continue into late adolescence, whereas hyperactivity symptoms decrease as they age^25^. Some cohort studies indicated that correlations between ADHD and its comorbidities may change along with participants’ expression of ADHD symptoms over time^26^,^27^. It reminds that the two ADHD symptoms should be discussed separately despite their combination forming ADHD as a syndrome; they may have distinct etiological determinants^28^,^29^. The second hypothesis is that relationships between ADHD symptoms and smoking/drinking are different in inattention and hyperactivity-impulsivity symptoms.

Furthermore, for clarifying the mediating effect of anxiety and depression on the relationship between ADHD symptoms and smoking/drinking, the interaction effect of ADHD symptoms and stressful life events on anxiety and depression needs to be discussed. People with ADHD may be vulnerable to stressful environment, as ADHD often comorbid with many emotional and behavioral problems, including anxiety and depression^5^. Stressful life events predict anxiety and depression in adolescent and young adult overtime and the effect is not moderated by genotype^30^. Thus, the third hypothesis is that stressful life events would strength the relationship between ADHD symptoms, and anxiety and depression by an interaction effect between stressful life events and ADHD.

## Results

Among a total of 1872 participations, 303 participants (16.19%) had the inattention sub-score ≥17, and 193 participants (10.31%) had the hyperactivity-impulsivity sub-score of the ASRS ≥17. There were 180 participants (9.62%) with a WURS score of 46 or above. These participants were placed into either ADHD symptomatic or non-symptomatic groups according to the criteria above, thus 7.75% of the sample were symptomatic, scoring above the cut-off point for ADHD. No significant gender difference was found between the two groups, even though male students showed a slightly higher rate of ADHD (8.78%) than female students (6.63%). Additionally, students in advanced grades showed increased rates of ADHD than those in lower grades. Table 1 shows the proportion of ADHD symptomatic and non-symptomatic participants, who smoked and drank, had anxiety, and depression. The ADHD symptomatic participants were significantly more likely to have a history of smoking and drinking, than ADHD non-symptomatic participants. The two groups also differed significantly in their behaviors in the last 30 days. 29.58% of ADHD symptomatic participants (versus 17.82% in comparison group) reported that they had smoked in the last 30 days, and 55.32% ADHD symptomatic participants (vs. 42.41% in comparing group) reported that they had drank alcohol in the last 30 days. Similarly, 52.82% ADHD symptomatic participants were intoxicated at least once in the past year; significantly higher than 29.65% in the comparison group. Participants in the ADHD symptomatic group had more anxiety symptoms than the non-symptomatic group. Similarly, 66.90% of participants in the ADHD symptomatic group had more depressive symptoms, significantly higher than 15.40% of non-symptomatic group.

**Figure 1:**
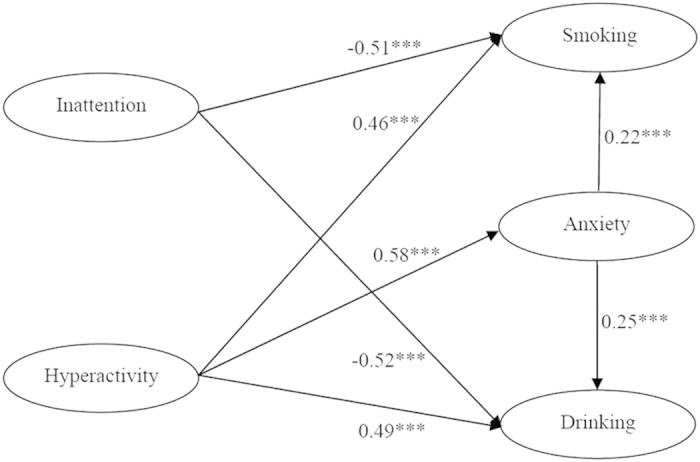
(SEM model 1). The relationship among ADHD symptoms, smoking/drinking and anxiety. ***P < 0.0001.

In Fig. 1 (SEM model 1), the unique outcomes were found with hyperactivity-impulsivity directly positively contributing to anxiety (*β* = 0.58, *P* < 0.001), smoking (*β* = 0.46, *P* < 0.001) and drinking behaviors (*β* = 0.49, *P* < 0.001). Meanwhile, the symptom of hyperactivity-impulsivity also indirectly predicted smoking (*β* = 0.13, *P* < 0.001) and drinking behaviors (*β* = 0.15, *P* < 0.001) through anxiety. It suggests that anxiety mediated hyperactivity-impulsivity and smoking/drinking behaviors, with the indirect effect of ADHD on smoking/drinking behaviors of 0.13 (0.58 × 0.22, *P* < 0.001) and 0.15 (0.58 × 0.25, *P* < 0.001), respectively. In contrast, inattention was found to negatively predict smoking (*β* = −0.51, *P* < 0.001) and drinking behaviors (β = −0.52, *P* < 0.001), and is not associated with anxiety. The proposed Model 1 represents an excellent fit to the data (NFI = 0.97, CFI = 0.97, & AGFI = 0.86). The loading factors for 9 observed variables of inattention range from 0.64 to 0.75, for 9 observed variables of hyperactivity was 0.68–0.75. The loading factors for 4 variables of smoking ranged from 0.62 to 0.89, for 10 variables of anxiety is 0.66–0.79, for 3 variables of drinking ranged from 0.53 to 0.86.

**Figure 2:**
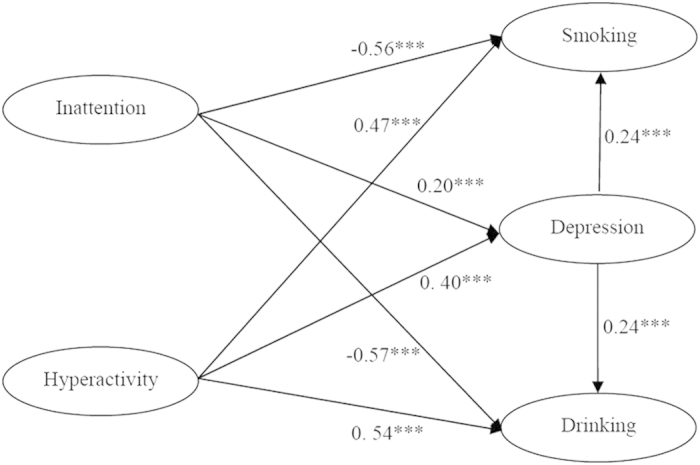
(SEM model 2). The relationship among ADHD symptoms, smoking/drinking and depression. ***P < 0.0001.

In Fig. 2 (SEM model 2), similar to Model 1, hyperactivity-impulsivity was directly and positively associated with smoking (*β* = 0.47, *P* < 0.001) and drinking behaviors (*β* = 0.54, *P* < 0.05). Alternatively inattention was negatively associated with smoking (*β* = −0.56, *P* < 0.001) and drinking (*β* = −0.57, *P* < 0.001). Both ADHD symptoms of hyperactivity-impulsivity (*β* = 0.40, *P* < 0.001) and inattention (*β* = 0.20, *P* < 0.001) were positively associated with depression. Depression also played an important role in mediating the effect pathway between ADHD symptoms and substance use. The indirect effect of hyperactivity-impulsivity on smoking and drinking behaviors was 0.1 (0.40 × 0.24 = 0.096, *P* < 0.001). The indirect effect of inattention on smoking and drinking behaviors was 0.05 (0.20 × 0.24 = 0.048, *P* < 0.001). Model 2 demonstrated an excellent fit to the data (NFI = 0.98, CFI = 0.98, & AGFI = 0.88). The loading factors for 9 observed variables of inattention range from 0.64 to 0.75, for 9 observed variables of hyperactivity was 0.68–0.75. The loading factors for 4 variables of smoking ranged from 0.60 to 0.91, for 13 variables of depression was 0.57–0.78, for 3 variables of drinking was 0.53–0.86.

The interactive effects of SLE and two ADHD core symptoms on anxiety and depression were shown in Table 2. Both ADHD symptoms of inattention (*β* = 0.152, *P* < 0.001) and hyperactivity-impulsivity (*β* = 0.363, *P* < 0.001), and SLE (*β* = 0.198, *P* < 0.001) significantly contributed to anxiety. Only the interactions between SLE and inattention symptoms significantly contributed to anxiety (*β* = 0.086, *P* < 0.05). Similar results were found for depression. To be specific, inattention (*β* = 0.226, *P* < 0.001), hyperactivity-impulsivity (*β* = 0.299, *P* < 0.001), and SLE (*β* = 0.2, *P* < 0.001) were significantly associated with depression. Only the interaction between SLE and inattentive symptoms significantly contributed to depression (*β* = 0.1, *P* = *P* < 0.05).

The *R*^*2*^ values in Table 2 showed increase in the variance explained by each successive model, with the final models explaining 32.8% and 33.8% of the variance for anxiety and depression respectively. Adding the interaction of SLE and inattention, and interaction of SLE and hyperactivity-impulsivity to the model (Block 3) increased the variance of 0.8% and 1% for anxiety and depression respectively. The regression models (Block 2 and 3) for anxiety and depression were strong, as indicated by the *F* values in Table 2.

## Discussion

No study has included mental health as mediator for assessing the effect of ADHD on substance abuse. Our study tested a conceptual model in which two ADHD symptom domains have different effect pathways on college students’ smoking and drinking behaviors, and found that their relationships may be mediated by the presence of anxiety or depression. The findings of the current study are consistent with previous studies showing adults have more prominent inattention (IN) than hyperactivity-impulsivity (HI) symptoms^24^. Furthermore, our study shows that HI symptoms are a more robust predictor of smoking and drinking behaviors among college students. This is supported by another non-clinical population-based study showing that hyperactivity-impulsivity is more strongly associated with a lifetime risk of smoking than inattentive symptoms^31^. This finding may be due to the fact that HI is closely related to a neuropsychology deficit of delay aversion, resulting in the high risk of substance use^32^.

Another interesting finding of our study is that two ADHD core symptoms have different associations with anxiety and depression. HI is closely related to both anxiety and depression, whereas IN is only related to depression. These associations are supported by a study showing increased episodes of major depression in the inattentive and combined ADHD subtype^33^. A history of ADHD in adolescence was associated with elevated risk of major depression through early adulthood, a relationship that remained significant after controlling for psychosocial impairment in adolescence and co-occurring psychiatric disorders^34^. This implies that only depression plays a crucial mediating role in the association between IN and substance use in college students. In another word, IN could predict smoking or drinking behaviors only in students with comorbid depression. New evidence found in the present study validated the hypothesis proposed in existing literature that depression is also commonly related to ADHD symptoms, and plays an important mediating role on substance use^35^.

Depression and IN often work closely to affect college student’s development. For example, a study carried out in the United States and China showed that both depression and IN were associated with college students’ adjustment, and that the association was cross-cultural and not specific to the United States^36^. A previous study also showed that when ADHD occurred with substance use disorder, it was associated with a more severe risk of depression^37^. Increased levels of major depression will increase the risk of using alcohol compared with drugs as primary substance of abuse. Comorbidity patterns differed between ADHD subtypes with increased major depression in the inattentive and combined subtype, increased antisocial personality disorder in the hyperactive/impulsive and combined subtypes^38^.

The above finding may explain the contradictory findings about the relationship between inattention and smoking/drinking in previous studies. The relationship between ADHD symptoms and smoking is complex^39^. Research using clinical samples indicates that individuals with ADHD smoke at rates that are significantly higher than those of the general population and/or nondiagnosed controls among both adults (41–42% vs. 26% for ADHD and non-ADHD, respectively)^40^. Both of IN and HI can significantly increase the likelihood of ever regular smoking in the sample of young adults^41^. Especially, the risk for smoking was more strongly associated with inattention symptoms than by the diagnosis of ADHD itself in a clinical sample^42^,^43^. Similar association is found between inattention and drinking, for example, it has been found that worsening inattention symptoms during adolescence were associated with higher levels of early adult binge drinking and marijuana use^44^. Inattention also relate with alcohol use among adolescent girls regardless of antisocial behavior, rather than boys^45^.

In contrast, two studies have found no difference in the number of cigarettes smoked or level of nicotine dependence in adult smokers with ADHD compared with non-ADHD control groups^46^,^47^, since the different effects of HI and IN may weaken the association. The high levels of IN did not predict smoking progression^48^. It also explain the negative associations between IN and smoking /drinking is found in this present study partially. The negative associations should be understood in the model only, which separated the effect of hyperactivity-impulsivity, and was mediated by internalizing disorders. Besides, externalizing disorders are known to be predictors of later substance use in people with ADHD, thus further study should take externalizing disorders into account as well.

In addition, except for the mediating effect of depression on the relationship between IN and smoking/drinking, the interaction between IN and SLE may explain the complex association partially. The current study found that IN may affect mental health through an interaction with SLE, no such interaction was found with HI. This may be because young adults with more symptoms of IN experience higher levels of stress, and are at greater risk of developing mental health problems^49^. The finding may interpret the robust effect of IN in the same case. Recent research in neuroscience found that the interaction between stress and 5-HTTLPR may render individuals vulnerable to broadly defined self-regulation problems and this mechanism is not only relevant for internalizing symptoms of anxiety and depression but also for ADHD symptoms in adolescents and young adults^50^.

Finally, the different findings across these studies may relate to the sampling strategies used. The separate domains of ADHD symptoms may be differentially related to outcomes among both clinical and nonclinical populations. The differences may also reflect variances in the relationship between ADHD symptoms and smoking at various points in the life-course. For instance, Burke *et al* employed an adolescent sample, whereas the study by Kollins *et al* was a cross-sectional analysis of smoking in a young adult sample^31^,^43^. Furthermore, previous studies have not taken the mediator of mental health into consideration. In summary, the findings of this population-based study indicate that IN and HI have shown variant associations between substance use and mental health. Even through college students show more symptoms in IN than HI, HI has a more robust association with college student’s substance use than IN. IN also plays an important role by interacting with SLE. Anxiety and depression may not only be associated with substance use behaviors directly, but also play an important mediating factor between ADHD symptoms and substance use, especially in relation to the level of depression in college students.

## Methods

### Participants

This study was approved by the medical ethics committee of Fudan University. The corresponding author confirms that this study was performed in accordance with the approved social experiments guidelines and regulations. Informed consent was obtained from all participants. The totals of 1872 college students were recruited from two high educational institutions in Shanghai, China. One of them is a high professional-technology college which is a three-year College, and students with relatively poor academic achievement in high school will enter this kind of college in China. Another one is a four-year comprehensive university, and students in this university had a relatively good academic achievement in high school. The method of stratified cluster sampling was used. Firstly, six majors were selected randomly from each educational institution; secondly, all grades of the six majors from the high professional-technology college (grade one to three) and the comprehensive university (grade one to four) were selected; lastly, students were recruited randomly from each grade, and they were asked to fill a questionnaire in a health care class by school doctors who were trained to collect the data. Finally, the participants included 980 males (52.38%) and 892 females (47.62%), and their ages ranged from 16 to 25 years with a mean age of 19.13 (SD = 1.30). There were only 39 (2.0%) students younger than 18 years old and 6 (0.32%) students older than 23 years old.

### Measures

All participants were asked to complete a questionnaire consisting of the instruments for assessing ADHD, anxiety, depression, and health risk behaviors including smoking and drinking alcohol. The specific instruments were as follows:

Current ADHD: We used the Chinese version of Adult ADHD Self-Report Scale (ASRS)^51^. The scale is an instrument consisting of the eighteen DSM-IV-TR criteria, developed in conjunction with revisions of the WHO Composite International Diagnostic Interview (CIDI)^52^. The 18 questions of the ASRS were generated from a pool of questions about the symptoms of ADHD typically expressed among patients with adult ADHD. The ASRS consisted of two subscales, inattention and hyperactivity-impulsivity, each containing nine items. Each item mapped onto one of the 18 DSM-IV symptoms of ADHD. Each subject was asked how often a symptom occurred over the past six months on a five-point Likert scale with 0 for never, 0 for rarely, 1 for sometimes, 2 for often, and 3 for very often^53^. Total score for each subscale ranged from 0 to 36. Individuals with a sum score on either subscale of 24 or greater were considered highly likely to have ADHD. Scores of 17–23 were classified likely and 0–16 unlikely to have ADHD^52^,^53^. Cronbach’s alpha coefficients for the present sample were .88 for the inattention items, and .88 for the hyperactive-impulsive items.

Childhood ADHD: This study used the Wender Utah Rating Scale (WURS) to assess Childhood ADHD. The WURS is a 25-item scale designed to retrospectively question childhood symptoms of ADHD^54^. Items were rated on a five-point Likert scale where 0–4 represented not at all or slightly, mildly, moderately, quite a bit, and very much. Cronbach’s alpha for the current sample was .94.

Anxiety and Depression: Symptom Checklist -90-Revised (SCL-90-R) was used to assess depression and anxiety. The SCL-90-R is a screening measure of general psychiatric symptomatology^55^. Two dimensions measuring anxiety (10 items) and depression (13 items) were used in the study. The Checklist followed a self-report format and students were asked to rate the severity of their symptoms over the past week on a 5-point scale ranging from 0 (not at all) to 4 (extremely). Cronbach’ s alpha coefficients for the current sample were .95 for the anxiety items, and .96 for the depression items.

Tobacco and alcohol use behaviors: Four items on the questionnaire were included regarding participants’ smoking experience and smoking frequency in the last 30 days, and onset of smoking to assess participators’ tobacco use. Similarly, five questions regarding participants’ use of alcohol in the past year; alcohol use over the last 30 days; and if they had been intoxicated in the past year, were asked to reflect participators’ behavior of alcohol use. For instance, “In the past 30 days, how many days have you drunk over 5 cups in two hours” was asked to figure out the alcohol use frequency. These items were selected from the National Health Risk Behavior Investigation Questionnaire for adolescents in China^56^.

Stressful life events: A 15-items scale was used to cover stressful life events that regarding family, school and personal problems in the last half year. The scale was revised based on the College Student’s Stressful Event Checklist^57^. For example, “The death of a close family member” was asked, with an answer “Yes” scored as 1, and “No” scored as 0. The total score ranged from 0 to 15, and it was used in statistical analysis. Cronbach’s alpha coefficients for the current sample was .82 for this15-items’ scale.

A participant was classified as having significant symptoms of ADHD if his /her score was greater than the clinical cut-off points on both the ASRS (a measure of current ADHD symptoms) and WURS (a retrospective childhood measure of ADHD symptoms). One necessary criterion to diagnose an adult with ADHD is that he/she should have the symptoms in childhood. For ASRS scores, individuals with sum score on either subscale of 17 or greater were considered likely to have ADHD for reducing false negative rate, because one more criterion was added to screen ADHD-symptomatic students in the current study. Ward, Wender, and Reimherr (1993) cited a cut-off score for the WURS of 46, thus participants with a score of 46 or above would be classified as having ADHD as a child^54^.

The total score of anxiety ranged from 0 to 40, and participants with scores over 20 were considered to have anxiety symptoms. The total score of depression ranged from 0 to 52, and the participants with scores over 26 were considered to have depression symptoms based on a study which suggested a new norm of SCL-90 for college student in China^58^,^59^.

### Statistical Analysis

The relationship between ADHD symptoms, anxiety and depression and smoking/drinking was analyzed by using Structural Equation Modeling (SEM) by LISREL 8.7. Considering symptoms displayed in college students are different in inattention and hyperactivity-impulsivity symptoms, the structural equation model is constructed with each symptom separately. For exploring the mediating effect of anxiety and depression, two independent structural equation models were performed, and the results are shown in Figs 1 and 2. The final models are displayed with the significant paths only. In both models, the factor loadings of the individual observed variables on the latent factor were strong, ranging from 0.53-0.86.

We also conducted hierarchical regression analyses by SPSS 17.0 to explore the predictors of anxiety and depression (Table 2). Because the moderator variable of stressful life events (SLE) and independent variables (inattention, and hyperactivity-impulsivity scores) are both continuous variables, hierarchical linear regression analyses were applied. Both moderator and independent variables were centralized by a *Z*-score. Analyses were carried out in three steps which are shown in the three blocks in Table 2. Firstly, we examined the effect of gender, age, and grade on anxiety and depression respectively. Secondly, we added the factors of SLE and two ADHD core symptoms to the regression model. Thirdly, we added interactive effects of SLE and ADHD symptoms to the model.

## Additional Information

**How to cite this article**: Tong, L. *et al.* Mediating effect of anxiety and depression on the relationship between Attention-deficit/hyperactivity disorder symptoms and smoking/drinking. *Sci. Rep.* **6**, 21609; doi: 10.1038/srep21609 (2016).

## Figures and Tables

**Table 1:** Characteristics of study populations with and without ADHD symptoms.

	*% (N)*	ADHD		*χ^2^*
		Symptomatic,*% (N)*	Non-symptomatic, *% (N)*	
Gender				
Male	52.4 (980)	8.8 (86)	91.2 (895)	3.028
Female	47.6 (892)	6.6 (59)	93.4 (832)	
Grade				
One	57.7(1079)	6.3 (68)	93.7 (1013)	
Two	25.6 (480)	8.1 (39)	91.9 (440)	15.7608**
Three	12.4 (233)	10.5 (25)	89.5 (213)	
Four	4.3 (80)	17.6 (13)	82.4 (61)	
Smoking (ever)				
Yes	38.6 (690)	53.6 (74)	37.3 (616)	14.3031***
Smoked last 30 days				
Yes	18.7 (346)	29.6 (42)	17.8 (304)	11.9094***
Smoking (more than one cigarette per day)				
Yes	8.6 (161)	14.5 (21)	8.1 (140)	6.9186**
Drinking (ever)				
Yes	78.4 (1444)	91.4 (127)	77.4 (1317)	14.8642***
Drank last 30 days (at least one drink one cup per day)				
Yes	56.5 (985)	55.3 (78)	42.4 (679)	8.7873**
Intoxicated at least once over the past year				
Yes	31. 6 (545)	52.8 (75)	29.6 (470)	43.3637***
Anxiety				
Yes	17.6 (329)	64.1 (93)	13.7 (236)	235.2426***
Depression				
Yes	19.4(363)	66.9 (97)	15.4 (266)	226.9267***
Total	100.0 (1872)	7.6 (145)	92.3(1727)	—

**p < 0.001, ***p < 0.0001.

**Table 2:** Hierarchical linear regression analysis of predictors of anxiety and depression.

	Block 1 Gender, grade and age	Block 2 SLE and ADHD	Block 3 SLE × ADHD
	*B*	*B*	*B*
Anxiety			
Gender	−0.023	0.009	0.004
Grade	0.065	0.023	0.025
Age	−0.027	0.022	0.018
Inattention		0.152***	0.149***
Hyperactivity-Impulsivity		0.363***	0.354***
SLE		0.198***	0.191***
SLE × Inattention			0.086*
SLE × Hyperactivity-Impulsivity			0.007
*R*^*2*^	0.002	0.320	0.328
*F*	1.902	128.227***	99.69
Depression			
Gender	−0.029	0	−0.005
Grade	0.062	0.026	0.027
Age	−0.018	0.026	0.022
Inattention		0.226***	0.224***
Hyperactivity-Impulsivity		0.299***	0.288***
SLE		0.200***	0.193***
SLE × Inattention			0.100*
SLE × Hyperactivity-Impulsivity			0.000
*R*^*2*^	0.002	0.328	0.338
*F*	2.208	132.349***	103.678***

SLE = Stressful life events; *P < 0.05, ***P < 0.0001.
